# Trend of Congenital Hypothyroidism Incidence and Its Affecting Factors in Shahr-e-Kord, Western Iran

**Published:** 2020-05

**Authors:** Moslem TAHERI SOODEJANI, Seyyed Mohammad TABATABAEI, Hamid Reza SHORAKA, Hosein FALLAHZADEH, Azimeh GHADERI

**Affiliations:** 1. Research Center of Prevention and Epidemiology of Non-Communicable Disease, Department of Biostatistics and Epidemiology, School of Public Health, Shahid Sadoughi University of Medical Sciences, Yazd, Iran; 2. Medical Informatics Department, School of Medicine, Mashhad University of Medical Sciences, Mashhad, Iran; 3. Vector-Borne Diseases Research Center, North Khorasan University of Medical Sciences, Bojnurd, Iran; 4. Shahr-e-Kord Health Services Center, Shahr-e-Kord, Iran

**Keywords:** Congenital hypothyroidism, Incidence, Trend, Risk factors, Predictors

## Abstract

**Background:**

Congenital hypothyroidism is one of the most common endocrine disrupters and metabolism, and is one of the most important preventable causes of physical and mental disabilities.

**Methods:**

This was a case-control study, in which 54468 infants were screened from 2006 to 2014 in Shahre-Kord, western Iran. To describe the data, central and dispersion indices such as mean and standard deviation was used. For modeling, logistic regression was used. All the tests were performed at the significant level of 5%.

**Results:**

Overall, 111 cases were diagnosed with hypothyroidism, which made the prevalence value equal to 2 cases per 1000 births. The prevalence in females and males was 1.9 and 2.2 per 1,000 birth, respectively. The odds ratio for this disorder was 4.47(2.42–9.28) for the neonates with a family history of hypothyroidism and 1.72(1.05–2.82) for those born through cesarean.

**Conclusion:**

The incidence of this disorder is similar in males and females, and the incidence of this disease in people with a family history is far more than others.

## Introduction

Congenital hypothyroidism is one of the most common endocrine disrupters and metabolism and is one of the most important preventable causes of physical and mental disabilities (1). According to the definition, hypothyroidism in newborns refers to deficiency of thyroxin during embryonic period and infancy (2). Such a situation, which is the deficiency of thyroxin (T4), in this period can lead to severe and irreversible damage to the brain in the absence of prompt and timely treatment (3). The main feature of this disease is the absence of specific symptoms so that most newborns seem normal at birth because of receiving maternal thyroxin through the umbilical cord (4).

Hypothyroidism in the fetus causes disturbances in important organs including the central nervous system and skeletal system, but most newborns seem quite natural at birth. Hypothyroidism is associated with complications such as mental retardation, shortness and hearing loss; since the brain development continues until about three years, the complications of the disease are irreparable in the absence of early diagnosis and early onset of the treatment (5).

According to Iranian hypothyroidism screening guideline Congenital hypothyroidism can be referred when Thyroid-Stimulating Hormone (TSH) is greater than 5 mu/l of heel sample in infants 3–5 d on filter paper (S&S 903 paper) and confirmation with venous blood samples TSH is greater than 10mu/l and thyroxin (T4) less than 6.5gr/dl (6). According to national studies, the prevalence of this disorder is higher than the global rate (7).

One of the possible causes of the incidence of congenital hypothyroidism in different parts of the world is iodine deficiency and the related disorders (8). In the areas where iodine deficiency is still common, the incidence of congenital hypothyroidism, particularly its transient type, is somewhat high (9).

Before the onset of neonatal screening programs, the incidence of congenital hypothyroidism, which can only be detected after the appearance of clinical symptoms of the disease, ranged from 1 in 7,000 to 1 per 10,000 live births (10). However, after starting the screening, the incidence of congenital hypothyroidism was reported initially in the range of 1 per 3,000 to 1 per 4,000 live births (11). The incidence of congenital hypothyroidism in male gender, in the Asiatic race, in children with severe underweight at birth (<1500 grams), multiple births and children of mothers over the age of 39 yr was higher, which confirmed the effect of some of the demographic factors on the incidence of this disease (12).

According to the high prevalence of this anomaly in western Iran, the aim of this study was to investigate the trend & effective factors of congenital hypothyroidism.

## Materials and Methods

### Study design and population

This was a case-control study. From 2006 to 2014, 54,468 neonates from Shahre-kord, western Iran were screened, of whom 111 had hypothyroidism and were considered as cases. The final diagnosis of this abnormality in neonates was carried out according to the national guidelines for neonatal hypothyroidism in Iran (13). According to the incidence of hypothyroidism, 222 neonates were extracted from the rest of the neonates as controls using simple random method and based on their record number. In each area where cases were identified, controls were also selected based on the gender ratio from the same area. Controls were matched based on the neonates’ gender and place of residence.

This study was confirmed by Ethics Committee of Shahid Sadoughi University of Medical Sciences. (Ethical code: IR.SSU.SPH.REC.1394.63).

Data gathering: Neonatal data such as weight and height at birth, gender and type of birth as well as parental data such as inter-family marriage and family history of the disease were gathered from their health records.

Data analysis: In order to describe the data, central and dispersion indices such as mean and standard deviation were used. Logistic regression was employed for modeling. All the tests were performed at a significant level of 5%. SPSS software (ver. 22, Chicago, IL, USA) was used to analyze the data.

## Results

Overall, 111 cases were diagnosed with hypothyroidism, which caused the prevalence to be 2 cases per 1000 births; the prevalence in girls and boys was 1.9 and 2.2 per 1,000, respectively.

**Fig. 1:**
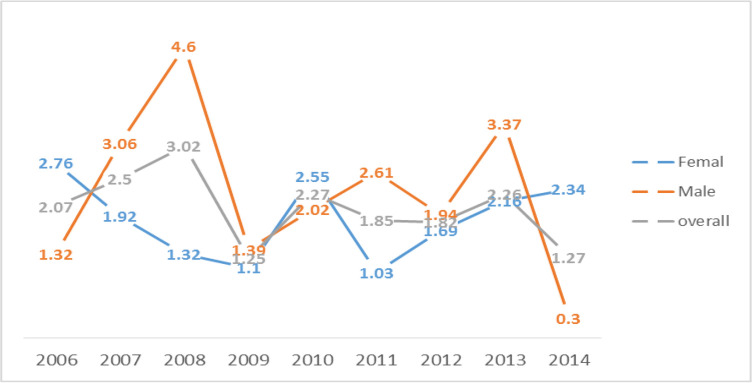
Prevalence of congenital hypothyroid disorder (per thousand births) during 2006–2014

The prevalence of this congenital disorder was not consistent with any particular structure and was observed as increased or decreased in both genders. However, in general, a relatively decreasing trend was observed in its overall prevalence during these years but this change was not significant (*P*=0.20) and, generally, the prevalence of males was relatively higher (Fig. 1).

Out of the 111 cases, 62 cases (56%) were male and the rest were female, also considered in controls.

The mean birth weight in cases was 2984 ± 540 and, in controls, it was 3,014 ± 440 gr. The height of the newborns was another variable investigated in this investigation. The mean height of the infants in the case group was 48.6 ± 3.3 and, in the control group, it was 49 ± 2.8 cm.

The primary mean of neonatal TSH in the case group was 23.7 ± 30.2 and, in the control group, it was 1.75 ± 2.2. Other characteristics of the case and control groups are listed in Table 1.

**Table 1:** Distribution of different factors among cases and controls

***Variable***		***Cases***		***Controls***	
		***Frequency***	***Percent***	***Frequency***	***Percent***
Gender	Female	49	44.1	100	45
	Male	62	55.9	122	55
Birth Weight	<2500 gr	24	26.1	29	13.1
	2500–4000gr	84	75.7	191	86
	4000gr<	3	2.7	2	0.90
Birth Seasonal	Spring	24	26.1	55	24.8
	Summer	36	32.4	62	27.9
	Autumn	28	25.2	54	24.3
	Winter	23	20.7	51	23
Family History	Yes	30	27	16	7.2
	No	81	73	206	92.8
Parental Consanguinity	Yes	31	27.9	55	24.8
	No	80	72.1	167	75.2
Delivery Type	Vaginal Delivery (NVD)	40	36	114	51.4
	Cesarean Section (C/S)	71	64	108	48.6

Logistic regression was used to investigate the predictor factors. At first, the analyses were performed as univariate analysis; then, multivariate analysis was employed to adjust the confounders (Table 2).

**Table 2:** Crude & adjusted predictors’ odds ratio in congenital hypothyroidism

***Predictors***		***Crude OR***	***Confidence Interval***	***P-value***	***Adjusted OR***	***Confidence Interval***	**P*-value***
Gender	Female	0.96	0.61–1.53	0.88	0.87	0.54–1.42	0.59
	Male	1	-		1	-	
Birth Weight	<2500gr	1.35	0.77–2.38	0.29	1.36	0.62–1.94	0.70
	2500–4000gr	1	-	-	1	-	-
	>4000	3.25	0.53–19.83	0.20	3.62	0.57–22.77	0.17
Family History	No	1	-	-	1	-	-
	Yes	4.76	2.47–9.22	<0.001	4.47	2.42–9.28	<0.001
Parental Consanguinity	No	1	-	-	1	-	-
	Yes	1.17	0.7–1.97	0.54	1.11	0.64–1.94	0.70
Delivery Type	Vaginal Delivery (NVD)	1	-	-	1	-	-
	Cesarean Section (C/S)	1.87	1.17–2.99	<0.01	1.72	1.05–2.82	0.03

In this part of the analysis, although the ratio of low weight in the case group was greater than the control group, there was no significant relation between LBW and CHD (congenital hypothyroid disorders). Moreover, it was similar for inter-family marriage, so that although this kind of marriage was more in the case group, this difference was not statistically significant.

The odds ratio of congenital hypothyroidism was higher for those who had a history of this disorder in their family, as the odds ratio for the ones who had the history of this disorder in their family was 4.47.

On the other hand, babies born with cesarean section were at risk for this disorder and the odds ratio was 1.72, which is statistically significant.

## Discussion

Since the prevalence of congenital hypothyroidism which may cause mental retardation in newborns is relatively high in Iran, understanding the risk factors of this disease can help to prevent and treat it (5, 14).

In Iran, various prevalence rates have been reported from 5 cases per 10,000 births to 18 cases per 1,000 births (15, 16). In this study, the overall prevalence was 2 cases per 1000 births, also observed in Isfahan and Qazvin provinces (17, 18). However, it was higher than the reported prevalence in Fars and Zanjan provinces (17, 19) and less than the reported prevalence in Khorasan Province (15).

It has been reported from one case per 4,000 births in Japan to 3 cases per 1,000 births in China (20, 21). This difference may be due to diet or genetic differences.

Studies on the relationship between the sex of the neonates and incidence of congenital hypothyroidism show that women are more likely to have this disorder than men (22). Although in our study, reverse results were found and men were more likely to have this disorder, this difference was not significant. This finding has happened in other studies as well (23). The prevalence of this disorder is the same in both sexes.

Infants are born in different seasons and their birth season may have an effect on the level of TSH; but, the result of our study was not significant as well as another study (14).

Another variable is birth weight, which has been a risk factor for the occurrence of congenital hypothyroidism in various studies. However, in the majority of studies, as well as the present one, the results have demonstrated no significant relationship between birth weight and anomalies (22–24). However, low birth weight could affect the level of TSH in neonatal babies (14, 25). Overall, weight cannot be considered as a factor in the prediction of congenital hypothyroidism.

Family history is considered as a risk factor in many congenital disorders. In the case of hypothyroidism, the results of our study, as well as others, confirm this relationship (23, 26). In cases with a family history of hypothyroidism, the re-occurrence of this malformation is likely to increase in that family.

Numerous inter-family marriages occur in Iran; hence, the possibility of occurrence of congenital disorders in the children of these parents is more than others. The results of this study showed that infants, the parents of whom had inter-family marriages, were more likely to have hypothyroidism than others, although this difference was not statistically significant (*P*=0.70). In other studies, significant results were obtained; for example, a study in Tehran Province showed that babies, the parents of whom had inter-family marriages, were 2.75 times more likely to have congenital hypothyroidism (27). Therefore, inter-family marriage seems to be a predictor of congenital hypothyroidism. Small sample size and high ratio of inter-family marriage in both case and control groups may have led this to be not statistically significant.

Several studies have investigated the effect of delivery type on the level of blood TSH, which has led to different results. In some works, higher prevalence of congenital hypothyroidism in neonates born with NVD is reported (14, 28). On the other hand, there are other studies that claim that the prevalence of congenital hypothyroidism in neonates born with C/S is higher (29). Although the results of our study were closer to the second claim, this difference was not statistically significant and the type of delivery seems not to be a predictor for congenital hypothyroidism.

## Conclusion

The incidence of this disorder is similar in males and females, and the occurrence of this disease in people with a family history is far more than others.

## Ethical considerations

Ethical issues (Including plagiarism, informed consent, misconduct, data fabrication and/or falsification, double publication and/or submission, redundancy, etc.) have been completely observed by the authors.

## References

[1] Agrawal P Philip R Saran S (2015). Congenital hypothyroidism. IJEM, 19(2): 221. 25729683 10.4103/2230-8210.131748 PMC4319261

[2] Taheri-Soodejani M Fallahzadeh H Lotfi MH (2017). Screening for congenital hypothyroidism in Shahr-e-Kord: prevalence and recall rate during 2006–2014. FEYZ, 20(6): 574–80

[3] Bernal J Guadaño-Ferraz A Morte B (2015). Thyroid hormone transporters—functions and clinical implications. Nat Rev Endocrinol, 11(7): 406 17. 25942657 10.1038/nrendo.2015.66

[4] LaFranchi SH (2011). Approach to the diagnosis and treatment of neonatal hypothyroidism. J Clin Endocrinol Metab, 96(10): 2959 67. 21976744 10.1210/jc.2011-1175

[5] Zeinalzadeh AH Talebi M (2012). Neonatal screening for congenital hypothyroidism in East Azerbaijan, Iran: the first report. J Med Screen, 19(3): 123 6. 23060475 10.1258/jms.2012.012024

[6] Goodarzi E Ghaderi E Khazaei S Alikhani A (2017). The prevalence of transient and permanent congenital hypothyroidism in infants of Kurdistan Province, Iran (2006–2014). Int J Pediatr, 5(2): 4309 18.

[7] Hashemipour M Hovsepian S Kelishadi R (2009). Permanent and transient congenital hypothyroidism in Isfahan–Iran. J Med Screen, 16(1): 11 6. 19349525 10.1258/jms.2009.008090

[8] Ford G LaFranchi SH (2014). Screening for congenital hypothyroidism: a worldwide view of strategies. Best Pract Res Clin Endocrinol Metab, 28(2): 175 87. 24629860 10.1016/j.beem.2013.05.008

[9] Fan X Chen S Qian J (2015). Incidence and interrelated factors in patients With congenital hypothyroidism as detected by newborn screening in Guangxi, China. Glob Pediatr Health, 2: 2333794X14567193 5. 10.1177/2333794X14567193 PMC4784601 27335934

[10] Rastogi MV LaFranchi SH (2010). Congenital hypothyroidism. Orphanet J Rare Dis, 5(1): 17. 20537182 10.1186/1750-1172-5-17 PMC2903524

[11] Polak M Legac I Vuillard E (2006). Congenital hyperthyroidism: the fetus as a patient. Horm Res, 65(5): 235 42. 16582565 10.1159/000092454

[12] Harris KB Pass KA (2007). Increase in congenital hypothyroidism in New York State and in the United States. Mol Genet Metab, 91(3): 268 77. 17512233 10.1016/j.ymgme.2007.03.012

[13] Yarahmadi S Ali Mohammadzadeh K Tabibi S Maleki M, editors (2011). Presenting mathematics model of cost-benefit calculation of screening for congenital hypothyroidism in Iran. Int Math Forum, 6(14): 681 97.

[14] Dalili S Rezvany SM Medghalchi A (2012). Congenital hypothyroidism: a review of the risk factors. Acta Med Iran, 50(11):735–9. 23292624

[15] Namakin K Sedighi E Sharifzadeh G Zardast M (2012). Prevalence of congenital hypothyroidism In South Khorasan province (2006–2010). JBUS, 19(2): 191 9.

[16] Haghshenas M Pasha YZ Ahmadpour-Kacho M Ghazanfari S (2012). Prevalence of permanent and transient congenital hypothyroidism in Babol City-Iran. Med Glas (Zenica), 9(2): 341 4. 22926374

[17] Safari F Karimzadeh T Mostafavi F Mahram M (2009). Screening of congenital hypothyroidism in Qazvin province (2006–2008). JQUMS, 12(4): 43 49.

[18] Hashemipour M Dehkordi EH Hovsepian S Amini M Hosseiny L (2010). Outcome of congenitally hypothyroid screening program in Isfahan: Iran from prevention to treatment. Int J Prev Med, 1(2): 92 97. 21566768 PMC3075477

[19] Majid V Saeideh M Ali N Zahra S (2011). High incidence and recall rate of congenital hypothyroidism in Zanjan province, a health problem or a study challenge? Int J Endocrinol Metab, 4(3): 338 342.

[20] Noori Shadkam M Jafarizadeh M Mirzaei M (2008). Prevalence of Congenital Hypothyroidism and Transient Increased Levels of TSH in Yazd Province. JSSU, 16(3): 315.

[21] Zhao D-H Shen Y Gong J-M Meng Y Su L Zhang X (2016). Newborn screening for congenital hypothyroidism in Henan province, China. Clin Chim Acta, 452: 58 60. 26522654 10.1016/j.cca.2015.10.030

[22] Abdelmoktader AM (2013). Risk factors for congenital hypothyroidism in Egypt: results of a population case-control study (2003–2010). Ann Saudi Med,33(3): 273. 23793431 10.5144/0256-4947.2013.273 PMC6078519

[23] Anjum A Afzal MF Iqbal SMJ Sultan MA Hanif A (2014). Congenital hypothyroidism in neonates. IJEM, 18(2): 213 216. 24741519 10.4103/2230-8210.129114 PMC3987273

[24] Medda E Olivieri A Stazi MA (2005). Risk factors for congenital hypothyroidism: results of a population case-control study (1997–2003). Eur J Endocrinol, 153(6): 765 73. 16322381 10.1530/eje.1.02048

[25] Bijarnia S Wilcken B Wiley VC (2011). Newborn screening for congenital hypothyroidism in very-low-birth-weight babies: the need for a second test. J Inherit Metab Dis, 34(3): 827 33. 21331666 10.1007/s10545-011-9286-8

[26] Simsek E Karabay M Kocabay K (2005). Neonatal screening for congenital hypothyroidism in West Black Sea area, Turkey. Int J Clin Pract, 59(3): 336 41. 15857333 10.1111/j.1742-1241.2004.00222.x

[27] Carvalho DDT Rocha DRTW Arbex AK (2016). Hypothyroidism in Childhood and Adolescence. Open J Endocr Metab Dis, 6(01): 72.

[28] Herbstman J Apelberg BJ Witter FR Panny S Goldman LR (2008). Maternal, infant, and delivery factors associated with neonatal thyroid hormone status. Thyroid, 18(1): 67 76. 18302520 10.1089/thy.2007.0180

[29] McElduff A McElduff P Wiley V Wilcken B (2005). Neonatal thyrotropin as measured in a congenital hypothyroidism screening program: influence of the mode of delivery. J Clin Endocrinol Metab, 90(12): 6361 3. 16144951 10.1210/jc.2005-0786

